# Integrated ultrasound-based radiomics and deep learning models in screening breast intraductal high-risk lesions or carcinoma: a multicenter retrospective study

**DOI:** 10.3389/fonc.2026.1705400

**Published:** 2026-03-26

**Authors:** Na Li, RuiJiao Chang, Bo Jiang, Xin He, FengSheng Li, YongXin Li, SanLi Guan, Jun Lian

**Affiliations:** 1 Department of Ultrasound, The 4th (Xing Yuan) Hospital of Yulin, Yulin, Shaanxi, China; 2 Department of Ultrasound of the General Hospital of Ningxia Medical University, Yinchuan, Ningxia Hui Autonomous Region, China; 3 Department of ultrasound, The First Medical Centre, Chinese PLA General Hospital, Beijing, China; 4 Department of Ultrasound, The Second Affiliated Hospital of Xi’an Jiaotong University, Xi’an, Shaanxi, China; 5 Department of Ultrasound of the Xi’an Gaoxin Hospital, Xi ‘an, Shaanxi, China; 6 School of Automation and Intelligence, Beijing Jiaotong University, Beijing, China; 7 Department of Thoracic Surgery, China Aerospace Science and Industry Corporation 731 Hospital, Beijing, China; 8 Department of Ultrasound, Ankang Central Hospital, Ankang, China

**Keywords:** breast intraductal lesion, deep learning, multicenter study, radiomics, ultrasound

## Abstract

**Purpose:**

The aim of this study was to explore the diagnostic performance of ultrasound (US)-based radiomics combined with deep learning (DL) in the screening of high-risk and malignant intraductal breast lesions.

**Methods:**

This multicenter retrospective study included patients with breast intraductal lesions from January 2022 to June 2024 from five hospitals in China. In the training set, conventional US images were segmented and radiomics features were extracted. After feature selection using least absolute shrinkage and selection operator (LASSO) regression, a radiomics model was developed using logistic regression, and the DL model was constructed based on ResNet-50. An integrated model was constructed by fusing the predicted probabilities from single models. The diagnostic performance of US, radiomics, DL, and integrated models was compared in the internal and external validation sets.

**Results:**

A total of 785 lesions were collected, including 486 benign lesions and 299 high-risk or malignant lesions. In the training set (520 lesions), the integrated model achieved superior performance (area under the curve (AUC) = 0.946 [0.923, 0.964]) to that of the US model (AUC = 0.774 [0.732, 0.816]; *p* < 0.001) and the DL model (AUC = 0.873 [0.841, 0.905]; *p* < 0.001). In the internal validation (130 lesions) and external validation sets (135 lesions), the integrated model achieved the best AUC (internal: 0.891 [0.825, 0.939], external: 0.861 [0.791, 0.914]) among all single models (*p* < 0.05). Among single models, in the training set, the radiomics model (AUC = 0.938 [0.919, 0.958]) outperformed both US (AUC = 0.774 [0.732, 0.816], *p* < 0.0001) and DL models (AUC = 0.873 [0.841, 0.905], *p* < 0.001). In the external validation set, the AUC of the radiomics model (AUC = 0.827 [0.760, 0.895]) was higher than that of the US model (AUC = 0.651 [0.564, 0.731], *p* = 0.011).

**Conclusion:**

The integrated radiomics and DL model demonstrated potential clinical value in screening the high-risk or malignant breast intraductal lesions.

## Introduction

Breast cancer is the most common malignant tumor in women and the second most common cause of cancer-related deaths (1). Duct-originating lesions account for over 80% of all breast lesions and are commonly observed in clinical practice (2). High-risk intraductal breast lesions may progress to malignancy, while ductal carcinoma *in situ* (DCIS), a form of pre-invasive breast cancer, may further develop into invasive carcinoma. Early identification of high-risk intraductal lesions or DCIS during screening, followed by appropriate clinical interventions, can significantly improve patient prognosis (3–5). Therefore, accurate differentiation of high-risk intraductal breast lesions and DCIS from benign lesions during early screening is crucial. However, because of the subtle imaging differences between intraductal lesions, accurately distinguishing them from benign lesions remains a significant challenge.

Intraductal high-risk lesions include atypical ductal hyperplasia (ADH), flat epithelial atypia (FEA), and intraductal papilloma with atypical hyperplasia (6), most of which require treatment through local excision. Breast intraductal malignant lesions include intraductal papillary carcinoma and DCIS. The clinical significance of DCIS with and without microinvasion remains controversial (7, 8). Moreover, identifying microinvasion through imaging remains challenging. Given that local excision remains the primary treatment strategy for these lesions (9), it is crucial to detect both high-risk and malignant intraductal lesions during screening. Therefore, we analyzed these lesions collectively.

Most of the high-risk and malignant breast intraductal lesions were detected by mammography screening, which is highly sensitive to microcalcifications. On contrast-enhanced magnetic resonance imaging (MRI), these lesions are typically characterized by clusters of ductal or segmental ring-like enhancement along the ducts (10). In contrast, conventional ultrasound showed limited sensitivity, often resulting in missed diagnoses (11). Despite its limitations, ultrasound remains the primary screening tool for breast examination, especially in Asian women with denser breast glands (12). Multimodal ultrasound techniques, such as elastography and contrast-enhanced ultrasound, have improved the diagnostic accuracy of DCIS; however, their widespread application in large-scale screening programs remains challenging. Furthermore, clinical studies on the use of gray-scale ultrasound for the differential diagnosis of these lesions are limited, with small sample sizes and low quality being common issues.

Radiomics and deep learning (DL) algorithms extract quantitative features from medical images, allowing for systematic and objective analysis to improve diagnostic accuracy (13). Currently, some studies have utilized DL models, such as convolutional neural networks, for the diagnosis of breast cancer (14, 15). However, despite these advancements, there are relatively few published clinical studies focusing on intraductal breast lesions. Therefore, we hypothesized that radiomics and a DL algorithm could enhance the diagnostic performance of ultrasound in evaluating intraductal breast lesions.

This study aimed to use multicenter data to establish and validate conventional ultrasound, radiomics, and DL models, as well as an integrated diagnostic model in order to compare their diagnostic performance.

## Materials and methods

### Study cohort

This study was a multicenter retrospective study involving five study centers: the Department of Ultrasound of the Second Affiliated Hospital of Xi’an Jiaotong University (institution 1); the Department of Ultrasound of the Fourth (Xing Yuan) Hospital of Yulin (institution 2); the Department of Ultrasound of the General Hospital of Ningxia Medical University (institution 3); the Department of Ultrasound of the Xi’an Gaoxin Hospital (institution 4); and the Department of Ultrasound of the First Medical Center of the Chinese PLA General Hospital (institution 5). This study was a retrospective study, and it obtained approval from the ethics committee of institution 1 (Ethics No. 2022136). The informed consent was waived because private information of patients was anonymized and omitted from the analysis.

**Figure 1:**
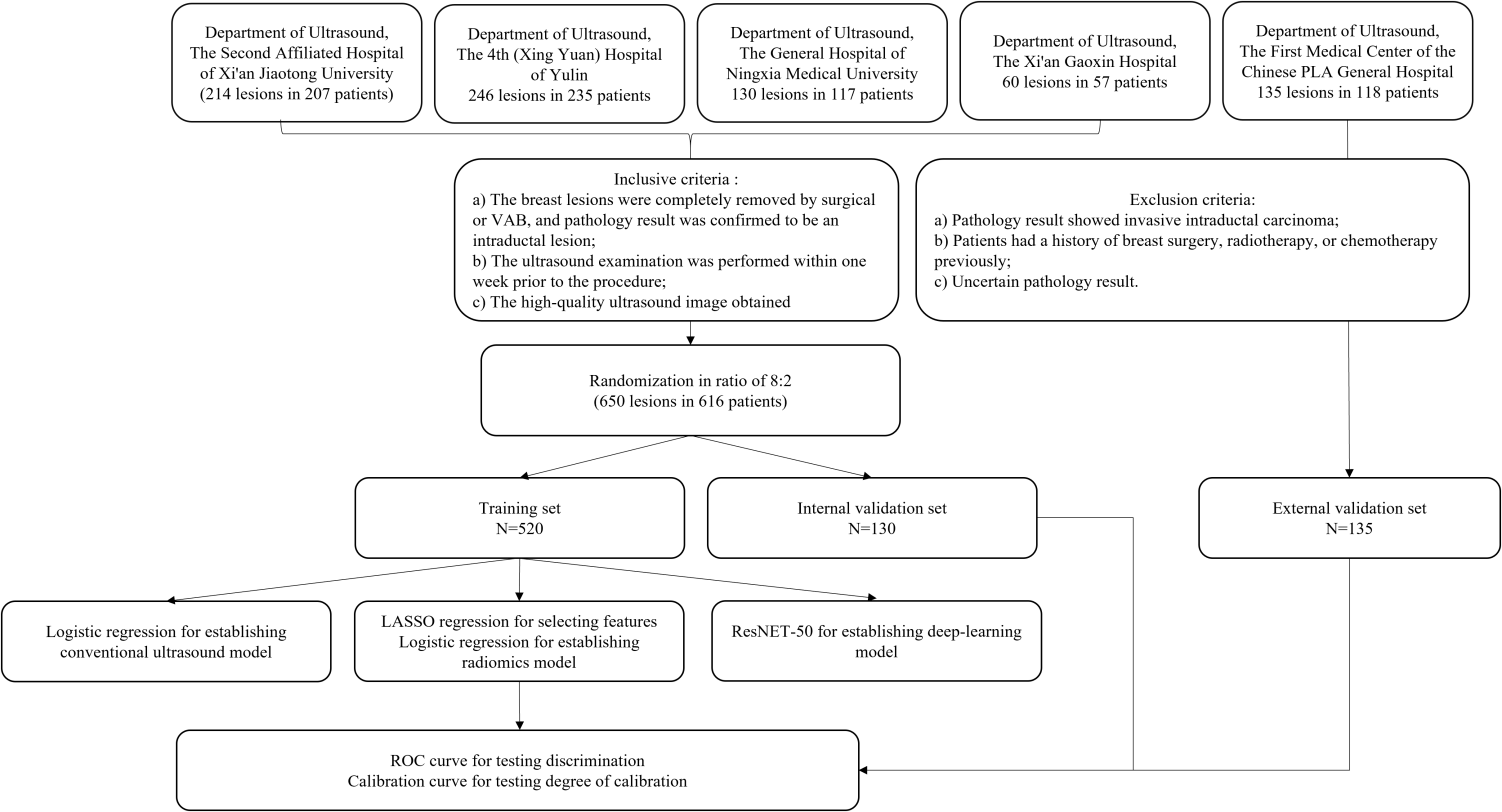
Flowchart depicting patient and lesion inclusion from fivehospitals, outlining inclusion and exclusion criteria, dataset randomization into training,internal validation, and external validation sets, and methods for establishing ultrasound,radiomics, and deep-learning models with ROC and calibration curve evaluation.

Patients with breast lesions who underwent surgical excision or complete ultrasound-guided vacuum-assisted biopsy (VAB) between January 2022 and June 2024 at the five study centers were included. The inclusion criteria were as follows: (a) the breast lesions were completely removed by surgical or VAB, and pathology result confirmed the lesions to be intraductal lesion; (b) the ultrasound examination was performed within 1 week prior to the procedure; and (c) high-quality ultrasound images were available. The exclusion criteria were as follows: (a) pathology results indicated invasive ductal carcinoma; (b) patients had a history of breast surgery, radiotherapy, or chemotherapy previously; and (c) inconclusive pathology result. The workflow of patient data collection is shown in **Figure 1**.

### Ultrasound images collection and analysis

All ultrasound examinations were performed by radiologists with at least 5 years of experience in breast ultrasound. Before the study, all participating radiologists underwent training in image quality screening and submitted 10 case images for assessment. The images were acquired using Doppler ultrasound equipment available at each center, including the following devices: Mindray Resona 7, Siemens S3000, Siemens ACUSON Sequoia, Philips Epiq 7, GE Logiq E9, GE Logiq E20, Hitachi ARIETTA 70, and Esaote MyLab 70, all equipped with high-frequency linear transducers. The image screening criteria included the following: (a) the lesion was fully displayed, and the image captured the section of the largest diameter of the lesion; (b) images were acquired using frequencies above 7 MHz, with high resolution, clear details, and no motion artifacts; (c) the gain, focus, and dynamic range were appropriately adjusted; (d) the deepest part of the lesion was positioned within 1/2 to 2/3 of the far field of the image; and (e) the focus was set at the same depth as the lesion. For each nodule, one image meeting these criteria was selected for radiomics feature extraction.

All ultrasound images were independently analyzed by two radiologists with over 10 years of experience in breast ultrasound diagnosis. The results were compared, and if the radiologists reached an agreement, the result was recorded as final. In cases of disagreement, the results were discussed and resolved by a supervising radiologist.

Conventional ultrasound features assessed included composition, echogenicity, homogeneity, margin, morphology, aspect ratio, microcalcifications, and the presence of peripheral ductal dilatation. Each lesion was categorized based on the 5th edition of the Breast Imaging Reporting and Data System (BI-RADS).

### Pathology examination and analysis

Pathology examinations were conducted by the pathology department at each study center. Initial diagnoses were made by junior pathologists, and all cases were subsequently reviewed and finalized by senior physicians with over 10 years of experience in breast pathology. All pathological examinations followed the 2022 consensus guidelines for the pathological diagnosis of DCIS of the breast (16).

### Tumor segment and radiomics feature extraction

**Figure 2:**
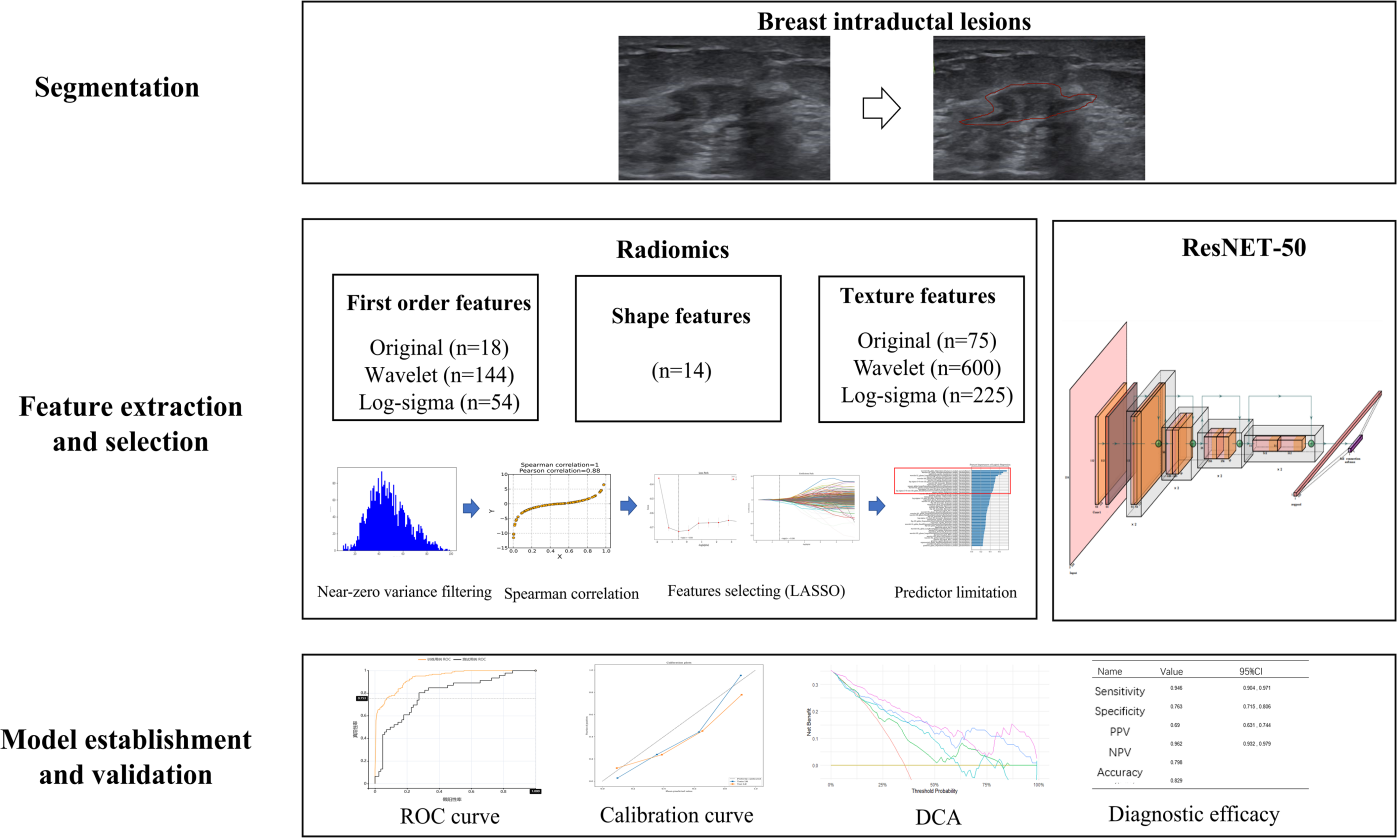
Flowchart depicting an artificial intelligence workflow for breast intraductal lesion analysis using ultrasound images, including segmentation, radiomics, feature selection methods, and ResNet-50 feature extraction followed by model establishment and validation with ROC, calibration, DCA curves, and diagnostic efficacy statistics.

The procedures for establishing and validating the radiomics and DL models are illustrated in **Figure 2**. Both segmentation and feature extraction of ultrasound images were performed using a web-based imaging radiomics research platform (Darwin Research Platform ver3.4.0: http://premium.darwin.yizhun-ai.com). For each lesion, the region of interest (ROI) was manually delineated on the gray-scale image showing the maximum lesion diameter by Radiologist A (5 years of experience in breast ultrasound) and then reviewed and adjusted by Radiologist B (10 years of experience). Both radiologists were blinded to the pathological diagnosis. To ensure radiomics reproducibility, inter-observer and intra-observer agreement of segmentation were assessed on a randomly selected subset of 50 lesions. Radiologist B independently re-segmented the same subset (inter-observer), and Radiologist A repeated the segmentation after a 2-week interval (intra-observer). Intraclass correlation coefficients (ICCs; two-way random-effects model, absolute agreement) were calculated for extracted radiomics features, and features with ICC < 0.75 were considered unstable and excluded from subsequent modeling.

### Radiomics feature selection

Radiomics feature extraction was performed on the ROI for each lesion. A total of 1,130 radiomics features were extracted from original and filtered ultrasound images, including first-order statistics, shape features, and texture features (e.g., GLCM, GLRLM, GLSZM, GLDM, and NGTDM). Prior to feature selection, all radiomics features were standardized using *z*-score normalization (subtracting the mean and dividing by the standard deviation) based on the training cohort. The same parameters were applied to the internal and external validation cohorts to prevent data leakage.

To reduce feature redundancy beyond least absolute shrinkage and selection operator (LASSO), a stepwise feature engineering workflow was applied. First, features with near-zero variance were removed. Second, pairwise correlation filtering was performed using Spearman correlation analysis; when two features had high correlation (|rho| > 0.90), one feature was removed to avoid multicollinearity. Finally, the remaining features were entered into LASSO regression with 10-fold cross-validation to select the optimal regularization parameter (lambda) and to identify features with non-zero coefficients. The selected radiomics features were used to build a model by multivariate logistic regression. We utilized stepwise regression in combination with the Akaike Information Criterion (AIC) to limit the final model to no greater than 18 features, ensuring compliance with the principle of a sample size greater than 10 events per variable (EPV). The obtained prediction probability served as the radiomics score (Rad score).

### Deep learning model

The DL model was developed using the ResNet-50 architecture with migration learning. The lesion ROI (based on manual segmentation) was cropped and resized to 224 × 224 pixels as network input. Pixel intensities were linearly rescaled to [0, 1]. Data augmentation (random rotation within ±15°, horizontal/vertical flipping, and random scaling) was applied to the training set to mitigate overfitting. A ResNet-50 model pre-trained on ImageNet was used as initialization. During training, the convolutional backbone was first frozen and only the final classification head was trained for 5 epochs; afterwards, the last residual block (layer 4) was unfrozen for fine-tuning. The network was trained for up to 30 epochs with a batch size of 16 using the Adam optimizer (initial learning rate, 1e−4; weight decay, 1e−5). A reduced LR on plateau learning-rate schedule and early stopping (patience = 5 epochs) were used. To address class imbalance, class-weighted cross-entropy loss was applied according to the inverse class frequency. The DL output probability was recorded as the DL score for subsequent integration.

### BI-RADS adjusted by radiomics and deep learning models

BI-RADS category 3 was defined as benign, while BI-RADS category 4a, 4b, and 4c were defined as malignant. The consistency and discrepancies between BI-RADS categorization and radiomics model in the diagnosis of benign and malignant lesions were analyzed. Additionally, the impact of radiomics and DL models on BI-RADS categorization upgrades and downgrades was explored.

### Establishment of the integrated model

A predictive model combining probability scores from the conventional ultrasound model, the Rad score derived from the radiomics model, and the DL score obtained from the DL model was established using multivariate logistic regression. This strategy represents late fusion at the score level (stacked generalization). Before model combination, continuous predictors (Rad score, DL score, and continuous clinical variables) were standardized using the mean and standard deviation of the training cohort, and the same transformation was applied to the validation cohorts. The Pearson correlation analysis was conducted on the output results of the three single models to be incorporated into the logistic regression. When the correlation coefficient |*r*| ≥ 0.8, it is generally considered that strong multicollinearity exists between variables. Indicators exhibiting strong multicollinearity will be excluded. Following the multicollinearity check, the integrated model will be established. The integrated model produced the final predicted probability of high-risk or malignant lesions.

### Statistical analysis

All statistical analyses were performed using SPSS (version 26.0) and RStudio (version 2022.2.2).

In the model training progress, cases from institutions 1, 2, 3, and 4 were mixed and randomly split into a training set and an internal validation set with a case ratio of 8:2. Cases from institution 5 were used as the external validation set. A conventional ultrasound model and the radiomics model were established by multivariate logistic regression. During model training, class imbalance was handled using class-weighting strategies where applicable.

In the validation progress, the receiver operating characteristic curve (ROC) and area under the ROC curve (AUROC), sensitivity, specificity, accuracy, and their and 95% confidence interval (CI) were calculated and compared between different models. Comparison between AUROCs was based on the Delong test. The calibration curve and Brier score were used to demonstrate the calibration degree of models. Decision curve analysis (DCA) was performed to test the actual clinical net benefit. A *p*-value of less than 0.05 was considered as statistically significant.

## Results

### Baseline of patients and lesions

A total of 785 lesions were collected from 734 female patients, with a mean age of 45.2 ± 12.6 years. Among these lesions, 486 were benign, 46 were high-risk lesions, and 251 were malignant. The maximum diameter was 1.9 ± 1.1 cm, with a median maximum diameter of 1.6 cm [interquartile range (IQR): 1.0, 2.6 cm]. The pathological distribution was as follows: 486 benign cases (61.9%), including 118 mammary duct ectasia cases (15.0%), 123 intraductal papilloma cases (15.7%), 71 usual ductal hyperplasia cases (9.0%), 27 periductal mastitis cases (3.4%), and 147 ductal cystic hyperplasia cases (18.7%); 46 borderline tumor cases (5.9%), including 11 intraductal papilloma with ADH cases (1.4%) and 35 ADH cases (4.5%); and 253 malignant tumor cases (32.2%), including 27 intraductal papillary carcinoma cases (3.4%), 152 DCIS cases (19.6%), and 72 DCIS with microinvasion cases (9.2%).

### Consistency analysis between groups

Institutions 1, 2, 3, and 4 had a total of 650 lesions. After random division at a ratio of 8:2, 520 lesions were allocated to the training set, and 130 lesions were assigned to the internal validation set. Institution 5, serving as the external validation set, included 135 lesions.

After random assignment, between the training set and the internal validation set, all parameters, including age (*t* = 0.521, *p* = 0.603), diameter (*Z* = 0.096, *p* = 0.923), tumor character (χ^2^ = 0.007, *p* = 0.935), composition (χ^2^ = 0.221, *p* = 0.638), homogeneity (χ^2^ = 0.558, *p* = 0.757), margin (χ^2^ = 0.382, *p* = 0.536), morphology (χ^2^ = 0.414, *p* = 0.520), aspect ratio (χ^2^ = 0.118, *p* = 0.732), microcalcification (χ^2^ = 1.480, *p* = 0.224), peripheral ductal dilatation (χ^2^ = 0.025, *p* = 0.874), and BI-RADS constitutive ratio (χ^2^ = 6.134, *p* = 0.105), did not show any statistically significant difference, while there were significant differences among the training set, internal validation set, and external validation set in parameters such as diameter, tumor character, composition, margin, and morphology, as detailed in **Table 1**.

**Table 1:** Baseline of patients and lesions in three groups.

Parameters	Categories	Training set (*n* = 520)	Internal validation set (*n* = 130)	External validation set *n* = 135)	Statistical value	*p*-value
Age (years)^*^	–	45.6 ± 12.4	44.9 ± 13.6	43.9 ± 12.3	*F* = 0.984	0.374
Diameter (cm)^φ^	–	1.5 (1.0, 2.4)	1.6 (1.0, 2.6)	2.0 (1.3, 3.1)	*F* = 14.555	0.001
Character	Benign,	334 (64.6)	84 (64.6)	68 (50.4)	χ^2^ = 9.215	0.010
	borderline, or malignant	186 (35.8)	46 (35.4)	67 (49.6)		
Component	Solid	452 (86.9)	115 (88.5)	129 (95.6)	χ^2^ = 7.951	0.019
	Solid-cystic	68 (13.1)	15 (11.5)	6 (4.4)		
Homogeneity	Homogeneous	218 (48.2)	52 (45.2)	65 (50.4)	χ^2^ = 8.627	0.071
	Heterogeneous	234 (51.8)	63 (54.8)	64 (49.6)		
Margin	Clear	177 (34.0)	48 (36.9)	25 (18.5)	χ^2^ = 13.743	0.001
	Unclear	343 (66.0)	82 (63.1)	110 (81.5)		
Morphology	Regular	65 (12.5)	19 (14.6)	1 (0.7)	χ^2^ = 17.663	<0.001
	Irregular	455 (87.5)	111 (85.4)	134 (99.3)		
Aspect ratio	>1	28 (5.4)	8 (6.2)	10 (7.4)	χ^2^ = 0.819	0.664
	≤1	492 (94.6)	122 (93.8)	125 (92.6)		
Microcalcification	Absent	439 (84.4)	104 (80.0)	114 (84.4)	χ^2^ = 1.558	0.459
	Present	81 (15.6)	26 (20.0)	21 (15.6)		
Ductal dilatation	Absent	296 (56.9)	75 (57.7)	63 (46.7)	χ^2^ = 4.925	0.085
	Present	224 (43.1)	55 (42.3)	72 (53.3)		
BI-RADS categories	3	158 (30.4)	40 (30.8)	21 (15.6)	χ^2^ = 22.599	0.001
	4a	288 (55.4)	61 (46.9)	89 (65.9)		
	4b	71 (13.7)	27 (20.8)	24 (17.8)		
	4c	3 (0.6)	2 (1.5)	1 (0.7)		

Categorical variables are expressed in number (percentage) and compared using the chi-square test.

^*^Variables are expressed in mean ± standard deviation and compared using the one-way ANOVA test.

^φ^Variables are expressed in median (interquartile range) and compared using the Kruskal–Wallis *H* test.

### Conventional ultrasound model

The multivariable logistic analysis of conventional ultrasound is shown in **Table 2**. Characteristics including older age (OR: 1.060, *p* < 0.001, 95% CI: 1.041–1.079), unclear margin (OR: 2.119, *p* = 0.003, 95% CI: 1.295–3.466), irregular morphology (OR: 3.496, *p* = 0.016, 95% CI: 1.260–9.697), and microcalcification (OR: 4.144, *p* < 0.001, 95% CI: 2.343–7.331) were the independent risk factors for malignant lesions. The conventional ultrasound model was built by these factors.

**Table 2:** The multivariable logistic regression for conventional ultrasound modeling.

Parameters	*B* value	SE	Wald	*p*-value	OR	95% CI	
Age (years)	0.058	0.009	42.067	<0.001	1.060	1.041	1.079
Diameter (cm)	0.019	0.102	0.035	0.852	1.019	0.834	1.246
Composition and echogenicity							
Solid-cystic	Reference						
Solid homogeneous hypoechoic	−1.33	0.332	0.161	0.688	0.875	0.457	1.677
Solid heterogeneous hypoechoic	−0.620	0.354	3.073	0.080	0.538	0.269	1.076
Margin (unclear)	0.751	0.251	8.937	0.003	2.119	1.295	3.466
Morphology (irregular)	1.252	0.520	5.783	0.016	3.496	1.260	9.697
Aspect ratio (>1)	0.251	0.466	0.290	0.590	1.286	0.515	3.206
Microcalcification	1.422	0.291	23.862	<0.001	4.144	2.343	7.331
Ductal dilatation	−0.030	0.235	0.016	0.899	0.971	0.613	1.538
Constant	−4.948	0.755	42.931	<0.001	0.007		

SE, standard error; OR, odds ratio; CI, confidence interval.

### Radiomics features selection and radiomics model establishment

**Figure 3:**
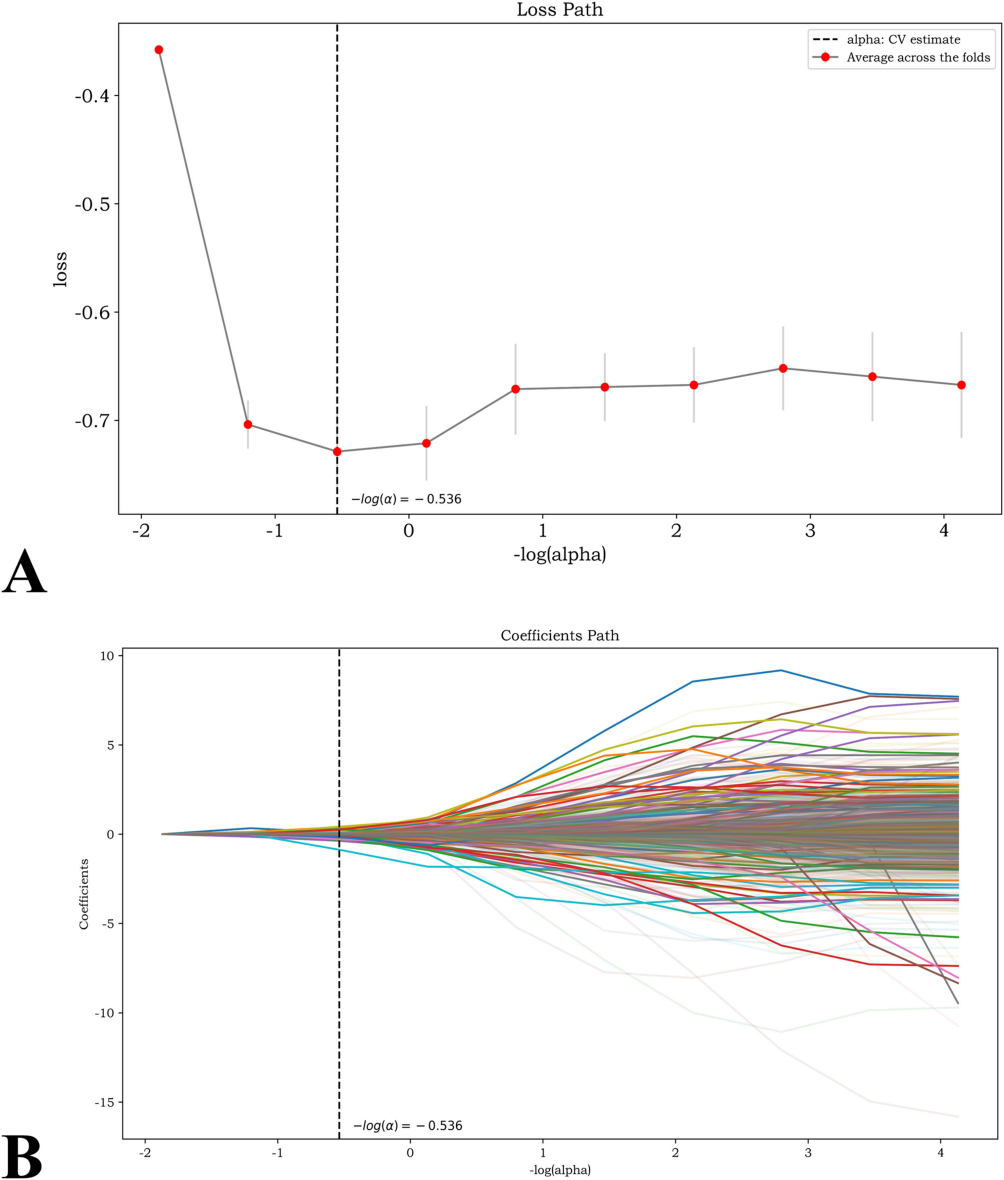
The radiomics feature selection progress. Panel A displays a line chart showing loss versus negative log alpha, with red dots for average loss across folds and a dashed vertical line indicating the cross-validated estimate at negative log alpha equals minus zero point five three six. Panel B shows a coefficients path plot across negative log alpha for numerous model coefficients, each in a different color, with the same vertical line marking the cross validated alpha estimate.

For the radiomics model, a total of 1,130 radiomics features were extracted. The regression coefficient path and coefficients of features selected from model are illustrated in **Figure 3**. After performing LASSO regression, 96 radiomics features were ultimately selected for modeling. In accordance with the AIC criterion, we limited the number of predictive variables in the final model to 18 after modeling. For segmentation reproducibility, the extracted radiomics features showed good agreement between observers and within the same observer. The inter-observer ICC and intra-observer ICC were both above the predefined threshold (ICC ≥ 0.75), indicating acceptable stability for radiomics analysis.

### Diagnostic performance

**Figure 4:**
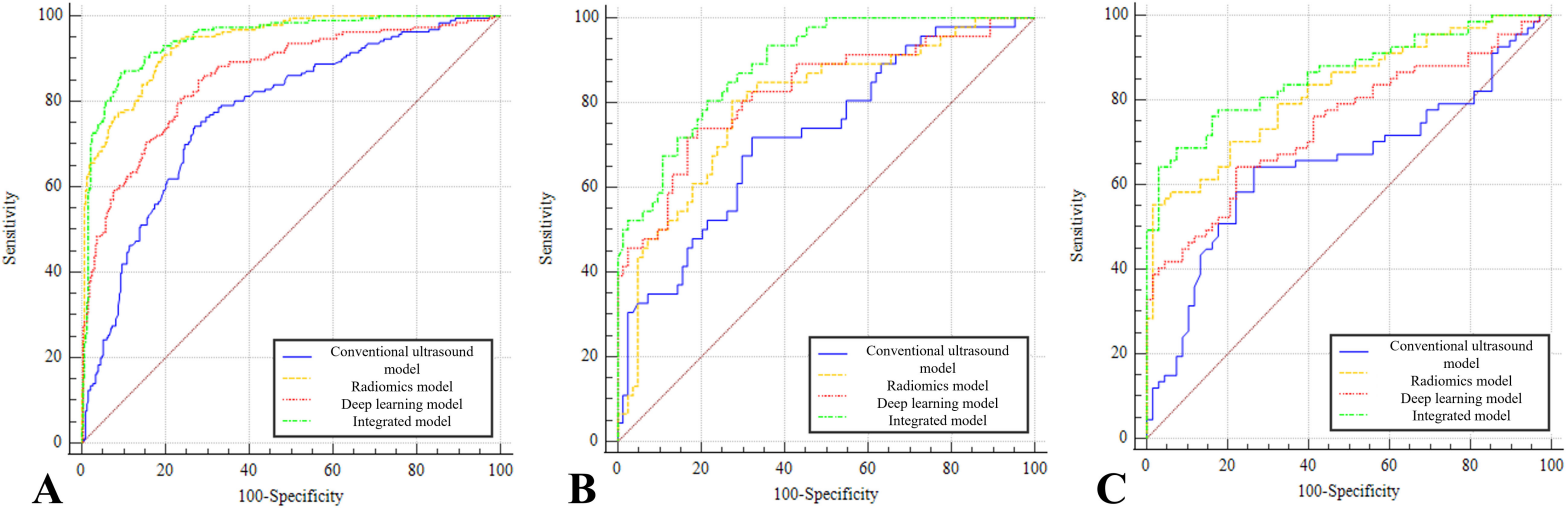
Receiver operating characteristic curves of different models. **(A)** Four ROC curves in the training set. **(B)** Four ROC curves in the internal validation set. **(C)** Four ROC curves in the external validation set. The lines of the integrated model (green line), the conventional ultrasound model (blue line), the deep learning model (red line), and the radiomics model (yellow line) are shown.

Before developing the integrated model, collinearity tests were conducted. No variables were excluded due to significant collinearity (correlation coefficients of 0.354, 0.450, and 0.597, respectively). The AUROC and diagnostic performance of each model in three cohorts are shown in **Table 3** and **Figure 4**. By comparison, in the training set, the AUROC of the integrated model was higher than that of the conventional ultrasound model (*Z* = 8.952, *p* < 0.0001) and the DL model (*Z* = 5.719, *p* < 0.0001). The AUROC of the radiomics model was higher than that of the conventional ultrasound model (*Z* = 7.491, *p* < 0.0001) and the DL model (*Z* = 4.884, *p* < 0.0001). The AUROC of the DL model was higher than that of the conventional ultrasound model (*Z* = 3.184, *p* = 0.0015). There was no statistically significant difference in diagnostic efficacy between the integrated model and the radiomics model (*Z* = 1.151, *p* = 0.249).

**Table 3:** Diagnostic performance of four models in the training, internal validation, and external validation sets.

Models	AUC	Accuracy	Sensitivity	Specificity	PPV	NPV	Brier score
Training set							
US model	0.774 (0.732, 0.816)	0.707 (0.667, 0.745)	0.742 (0.673, 0803)	0.731 (0.680, 0.778)	0.556 (0.512, 0.600)	0.836 (0.798, 0.868)	0.181 (0.165, 0.197)
Radiomics model	0.938 (0.919, 0.958)	0.829 (0.794, 0.860)	0.946 (0.904, 0.971)	0.763 (0.715, 0.806)	0.690 (0.631, 0.744)	0.962 (0.932, 0.979)	0.104 (0.090, 0.117)
Deep learning model	0.873 (0.841, 0.905)	0.802 (0.728, 0.803)	0.783 (0.718, 0.836)	0.813 (0.767, 0.851)	0.696 (0.630, 0.754)	0.872(0.831, 0.905)	0.141 (0.123, 0.159)
Integrated model	0.946 (0.923, 0.964)	0.892 (0.862, 0.917)	0.866 (0.808, 0.911)	0.907 (0.870, 0.936)	0.838 (0.787, 0.879)	0.923 (0.893, 0.945)	0.082 (0.068, 0.099)
Internal validation set							
US model	0.723 (0.638, 0.798)	0.692 (0.605, 0.770)	0.717 (0.565, 0.840)	0.679 (0.568, 0.776)	0.550 (0.460, 0.637)	0.814 (0.730, 0.877)	0.196 (0.162, 0.234)
Radiomics model	0.795 (0.713, 0.878)	0.754 (0.671, 0.825)	0.804 (0.668, 0.893)	0.726 (0.623, 0.810)	0.617 (0.490, 0.729)	0.871 (0.773, 0.931)	0.187 (0.144, 0.231)
Deep learning model	0.770 (0.680, 0.861)	0.769 (0.712, 0.858)	0.688 (0.657, 0.801)	0.817 (0.720, 0.886)	0.688 (0.547, 0.801)	0.817 (0.720, 0.886)	0.151 (0.116, 0.189)
Integrated model	0.891 (0.825, 0.939)	0.792 (0.712, 0.858)	0.804 (0.660, 0.906)	0.785 (0.682, 0.867)	0.672 (0.571, 0.760)	0.880 (0.801, 0.930)	0.135 (0.096, 0.176)
External validation set							
US model	0.651 (0.564, 0.731)	0.689 (0.604, 0.766)	0.642 (0.515, 0.755)	0.735 (0.614, 0.835)	0.705 (0.607, 0.787)	0.676 (0.595, 0.747)	0.237 (0.203, 0.276)
Radiomics model	0.827 (0.760, 0.895)	0.756 (0.674, 0.825)	0.544 (0.427, 0.657)	0.970 (0.898, 0.992)	0.949 (0.831, 0.986)	0.677 (0.578, 0.762)	0.172 (0.138, 0.205)
Deep learning model	0.756 (0.669, 0.842)	0.731 (0.626, 0.786)	0.625 (0.484, 0.748)	0.793 (0.693, 0.866)	0.638 (0.495, 0.760)	0.783 (0.683, 0.858)	0.201 (0.168, 0.236)
Integrated model	0.861 (0.791, 0.914)	0.807 (0.730, 0.870)	0.686 (0.561, 0.794)	0.926 (0.836, 0.975)	0.901 (0.795, 0.955)	0.750 (0.676, 0.811)	0.147 (0.107, 0.187)

Variables are expressed in value (95% confidence interval). AUC, area under the curve; PPV, positive predictive value; NPV, negative predictive value; US, conventional ultrasound.

In the internal validation set, the AUROC of the integrated model was higher than that of the clinically conventional ultrasound model (*Z* = 3.671, *p* = 0.0002), radiomics model (*Z* = 3.964, *p* = 0.0001), and DL model (*Z* = 5.719, *p* = 0.0462). The difference between the remaining models was not statistically significant (*p* > 0.05).

In the external validation set, the AUROC of the integrated model was higher than that of the conventional ultrasound model (*Z* = 4.477, *p* < 0.0001), radiomics model (*Z* = 2.249, *p* = 0.0245), and DL model (*Z* = 3.113, *p* = 0.0019). The AUROC of the radiomics model was higher than that of the conventional ultrasound model (*Z* = 3.253, *p* = 0.0011). The difference between the remaining models was not statistically significant (*p* > 0.05). The comparison details are shown in **Table 4**.

**Table 4:** Comparison of the area under the receiver operating characteristic curve among different models.

Comparison of AUROC	Training set		Internal validation set		External validation set	
	*Z* value	*p*-value	*Z* value	*p*-value	*Z* value	*p*-value
Inte vs. DL	5.719	< 0.001	1.994	0.046	3.113	0.002
Inte vs. RAD	1.151	0.249	3.964	< 0.001	2.249	0.025
Inte vs. US	8.952	< 0.001	3.671	< 0.001	4.477	< 0.001
RAD vs. DL	4.884	< 0.001	0.660	0.509	1.890	0.059
RAD vs. US	7.491	< 0.001	1.186	0.236	3.253	0.001
DL vs. US	3.184	0.002	1.711	0.087	1.469	0.1419

Use the Delong test for the comparison. Inte, integrated model; RAD, radiomics model; DL, deep learning model; US, conventional ultrasound model; AUROC, area under the receiver operating characteristic curve.

### Calibration performance and clinical decision analysis

**Figure 5:**
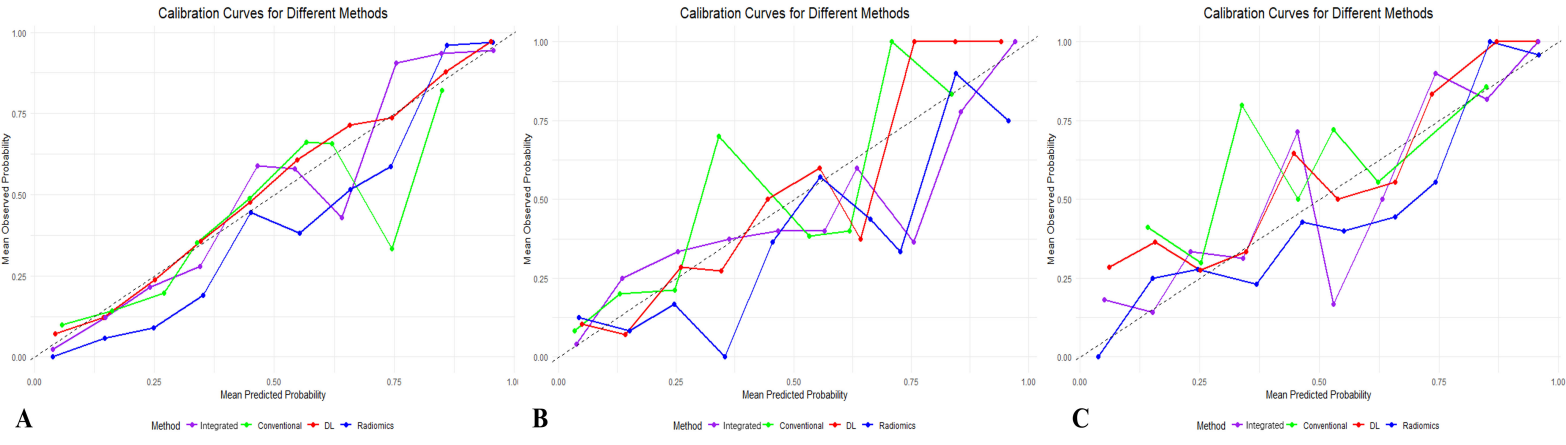
Calibration curves of different models. **(A)** Calibration curves of the four models in the training set. **(B)** Calibration curves of the four models in the internal validation set. **(C)** Calibration curves of the four models in the external validation set. The lines of the integrated model (purple line), the conventional ultrasound model (green line), the deep learning model (red line), and the radiomics model (blue line) are shown.

As shown in **Table 3**, compared to each single model, the integrated model showed better calibration tendency, with a Brier score of 0.082, 0.135, and 0.147 in the training, internal validation, and external validation sets. Among the single models, the radiomics model demonstrated the least Brier score of 0.104 and 0.172 in the training and external validation sets, respectively. In the internal validation set, the DL model showed the least Brier score of 0.151. The calibration curves of four models in each cohort are shown in **Figure 5**.

**Figure 6:**
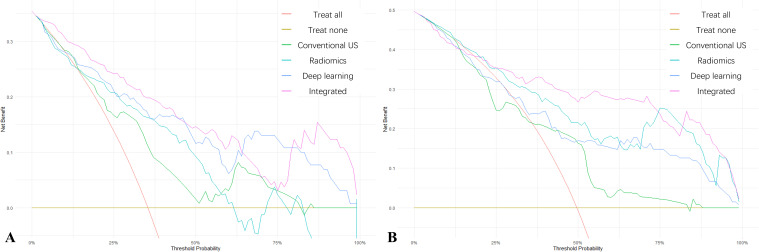
Decision curve analysis of different models. **(A)** Decision curves of the four models in the internal validation set. **(B)** Decision curves of the four models in the external validation set. The lines to diagnose all patients (red line) and to diagnose none of the patients (yellow line), and the lines of the conventional ultrasound model (green line), the radiomics model (light blue line), the deep learning model (dark blue line), and the integrated model (purple line) are shown.

**Figure 6** shows the clinical decision analysis curves of different models in the internal test set and the external test set. The results all demonstrate that compared with other single models, the integrated model had a more significant clinical net benefit.

### Integrated model for BI-RADS category adjustment

We complied with the guidelines by defining lesions in BI-RADS category 3 as “benign lesions” and lesions in BI-RADS categories 4a, 4b, and 4c as “suspicious lesions”. The proportion of cases correctly diagnosed by both the integrated model and BI-RADS was 44.8% (352/785), while the proportion of cases incorrectly diagnosed by both was 5.4% (42/785). The integrated model corrected the BI-RADS diagnosis in 49.8% (391/785) of cases, including 28 upgraded lesions and 363 downgraded lesions. In particular, the integrated model achieved a correct upgrade in 89.3% (25/28) of all upgrade cases and a correct downgrade in 80.4% (292/363) of all downgrade cases. The overall correct adjustment rate of the integrated model for BI-RADS was 81.1% (317/391), while the incorrect adjustment rate was 18.9% (74/391). The details of correlation between the integrated model and BI-RADS are presented in **Table 5**.

**Table 5:** The value of the integrated model for BI-RADS category adjustment.

	Integrated model		
	Diagnosed as benign	Diagnosed as malignant	Total
Malignant in pathology	95	204	299
BI-RADS 3 categories	24	25	49
BI-RADS 4a, 4b, 4c categories	71	179	250
Benign in pathology	465	21	486
BI-RADS 3 categories	173	3	441
BI-RADS 4a, 4b, 4c categories	292	18	45

Categorical variables are expressed in number.

## Discussion

In this study, we developed and validated an ultrasound radiomics model, a DL model, and an integrated model to screen high-risk or malignant intraductal breast lesions by analyzing quantitative features extracted from ultrasound images. All models significantly improved the diagnosis of intraductal lesions compared to conventional ultrasound features, with the integrated model demonstrating the best diagnostic efficacy.

Invasive ductal carcinoma of the breast tends to exhibit apparent malignant features on conventional ultrasound imaging, resulting in a relatively low misdiagnosis rate compared to intraductal lesions (17). In contrast, intraductal breast lesions are confined to the ducts and do not exceed the basement membrane. High-risk intraductal breast lesions and intraductal carcinoma represent early stages of malignant disease, making differentiation from benign lesions during screening particularly challenging and prone to misdiagnosis (18, 19). Even in cases with microinvasion, detection on imaging remains difficult due to the depth of infiltration being less than 1 mm (7). In this study, conventional ultrasound features, including unclear margin, irregular morphology, and microcalcifications were identified as independent risk factors for high-risk or malignant lesions. This is in accordance with the malignant features of BI-RADS. In contrast, the taller-than-wide orientation was not included as a risk factor, which may be explained by the fact that the lesion had a distended lateral growth along the ductal lumen and had not yet broken through the basement membrane to form an infiltrative growth pattern (20, 21). The diagnostic efficacy remains unsatisfactory after conventional ultrasound modeling. Therefore, we applied radiomics and DL techniques to improve screening accuracy so that active surveillance or necessary biopsy of suspicious lesions can be performed timely.

Our study demonstrated that incorporating radiomics and DL can significantly enhance the diagnostic performance of conventional ultrasound. The integrated model achieved the best performance, with an area under the curve (AUC) exceeding 0.85 in both internal and external validation sets. This superior performance may be attributed to the use of a broader and more diverse imaging dataset. There were few previous studies that combine radiomics and DL to diagnose breast intraductal tumors. Wu used the integrated model to predict the nuclear grading of breast intraductal cancer, which was found to be more advantageous than a single model (22), which was similar to our findings. Meanwhile, previous studies have also developed machine learning and DL diagnostic models, but their AUC values were less than ideal (23). The model is likely influenced by factors such as sample homogeneity across research centers, variations in model parameters, and differences in lesion size distribution (24). We plan to conduct further research in the future to explore the potential impact of these factors on the model. Therefore, the establishment of the combined model may have a better prospect in the future. Considering the relatively limited sample size and the need for interpretability and robust calibration in clinical practice, we implemented score-level late fusion using multivariate logistic regression. More complex ensemble methods or late-fusion neural networks may further improve performance and will be explored in future studies.

In the single models, we found that both the radiomics and DL models showed a trend of having higher AUCs compared to the traditional ultrasound model, but no statistically significant differences were observed. These may be due to the fact that the sample size of the present study was relatively insufficient in the validation sets. Zhang et al. distinguished invasive ductal carcinoma from DCIS by combining intra- and peri-tumor features, achieving a diagnostic AUC of approximately 0.7 using 454 patients (24). In contrast, Yin et al. utilized a DL model for ultrasound to differentiate between DCIS and fibroadenoma of the breast, achieving an efficacy with an AUC of approximately 0.8 from 1,112 cases (23). In our study, the radiomics model achieves an efficacy of approximately 0.8 in both validation sets, while DL achieves an efficacy of approximately 0.75; thus, the relatively limited dataset size may partly explain why the radiomics model showed competitive performance compared with the DL model.

In all cohorts, the integrated model demonstrated higher sensitivity and specificity compared to the conventional ultrasound model, indicating superior reproducibility and generalizability. However, in the external validation set, the sensitivity of all models decreased compared to the training and internal validation sets. This variation in performance may be attributed to differences in the validation population, a common challenge faced by current clinical prediction models (25). To address this limitation and further improve the diagnostic efficacy of such models, multimodal imaging technology techniques, such as combining ultrasonography and elastography, could be considered for future development.

The results demonstrated that the integrated model effectively refined the ultrasound BI-RADS system. Intraductal breast lesions often present challenges for the BI-RADS system because they grow along the ducts and do not frequently exhibit typical malignant features, such as aspect ratios greater than one, irregular morphology, or unclear borders (20). Therefore, the application of radiomics and DL models has the potential to provide additional diagnostic information and improve diagnostic efficacy. In this study, most of the “pseudo-benign” lesions that were correctly upgraded by the integrated model only exhibited mildly irregular morphological characteristics. The application of the integrated model allowed 89.3% of these lesions to be correctly upgraded, avoiding missed diagnoses. Meanwhile, the majority of intraductal lesions (55% in this study) were classified as BI-RADS 4a. However, most of these were pathologically benign lesions, which were nonetheless suspected of malignancy, often leading to further examinations or unnecessary biopsies. The integrated model demonstrated the ability to reasonably downgrade these lesions, correctly downgrading 84.9% of benign BI-RADS 4 lesions, thereby reducing the likelihood of overdiagnosis and unnecessary interventions.

Although our study was the largest multicenter clinical study to date both using radiomics and DL for identifying intraductal breast lesions, there were several limitations. Firstly, the radiomics analysis was performed solely on gray-scale ultrasound images, without incorporating other modalities such as blood flow, elasticity, or microcirculatory perfusion of the lesions. Secondly, we focused on binary classification (benign vs. high-risk/malignant) and did not separately analyze high-risk lesions, malignant lesions *in situ*, and malignant lesions with microinvasion; future studies will address multi-class classification. Thirdly, although migration learning, data augmentation, and external validation were used to mitigate overfitting, the training sample size is still relatively limited for DL, and additional larger prospective datasets are warranted. Meanwhile, we did not include an analysis of the interpretability of DL to address the “black box” issue. Finally, we did not compare the ultrasound modality with other imaging techniques, such as mammography or MRI, which could have enhanced the persuasiveness of our findings.

In conclusion, integrated radiomics and DL models outperformed conventional ultrasound in identifying high-risk and malignant lesions from benign intraductal lesions in breast screening examination. This approach has the potential to provide a valuable support in clinical decision-making.

## Data Availability

The original contributions presented in the study are included in the article/supplementary material. Further inquiries can be directed to the corresponding authors.
